# 2466 MicroRNA-451: A potential key player in the development of diabetic nephropathy in an insulin resistant mouse model

**DOI:** 10.1017/cts.2018.95

**Published:** 2018-11-21

**Authors:** Maurice B Fluitt, Lijun Li, Narayan Shivapurkar, Carolyn Ecelbarger

**Affiliations:** Georgetown University

## Abstract

OBJECTIVES/SPECIFIC AIMS: MicroRNAs (miRNA) affect transcription of a number of genes involved in the development and progression of diabetic nephropathy (DN), and have become attractive therapeutic targets and biomarkers. Elevated renal gluconeogenesis, fibrosis, and albuminuria are early markers of incipient DN. Recent studies report that renal miRNA-451 may protect against DN and reduce renal gluconeogenesis in rodent models. MiRNA-451 is thought to act by targeting select factors resulting from disrupted insulin and growth factor signaling and the mechanistic-target of rapamycin (mTOR) in early DN. This study aimed to elucidate the role of miRNA-451 in the development and progression of DN. METHODS/STUDY POPULATION: To further elucidate the role of miRNA-451 in DN, we placed male insulin-resistant, TALLYHO/Jng mice on a high-fat diet (60% kCal). The mice were divided into 2 treatment groups and received 8 consecutive weekly intraperitoneal injections of locked nucleic acid (LNA) miR-451-inhibitor or LNA-scrambled compound (2 mg/kg·bw; n=8/treatment). Mice were euthanized after 12 weeks (4 weeks sans injections) and kidneys, liver, pancreas and abdominal adipose tissue were harvested for analysis. RESULTS/ANTICIPATED RESULTS: Renal homogenate expression of miRNA-451 was drastically reduced in inhibitor-treated mice (~6-fold) in comparison with scramble-treated mice. Western blotting of cortex homogenates for indicators of fibrosis and targets of miRNA-451 revealed a significant reduction in collagen IV (marker of epithelial integrity) in inhibitor-treated mice. In addition, metalloproteinase type 9 (MMP9, a known type IV collagenase), YWHAZ (a scaffolding protein and known target of miR-451), mTOR, and fructose bisphosphatase (FBP1, a rate-limiting gene in gluconeogenesis) were significantly increased in this group in comparison to scramble-treated mice. However, no differences were found in protein levels for glucose-6-phosphatase (G-6-Pase) or phosphoenolpyruvate (PEPCK), 2 additional gluconeogenic rate-limiting genes. MiRNA-451 antagonist did not significantly affect final body weight or blood glucose; however, mean blood sodium concentrations were slightly, but significantly higher (2%) in the LNA-inhibitor treated group (when compared with the scramble-treated group). No differences in blood potassium or chloride were found. Anion gap was 90% higher in the LNA-inhibitor treated group when compared with scramble-treated mice. No differences in urinary albumin to creatinine ratio were found between the two treatment groups. However, Masson Trichrome scoring revealed a 59% increase in fibrosis in inhibitor-treated mice. DISCUSSION/SIGNIFICANCE OF IMPACT: Collectively, these findings support a potentially protective role of miRNA-451 in attenuating signaling via mTOR that may alter both renal gluconeogenic potential (contributing to the diabetic phenotype) and activation and progression of renal fibrosis. Therapies to enhance miRNA-451 signaling may be beneficial to reduce renal pathology associated with DN.

